# Effects of practitioner’s experience on the clinical performance of ultrasound-guided central venous catheterization: a randomized trial

**DOI:** 10.1038/s41598-021-86322-y

**Published:** 2021-03-24

**Authors:** Hyun-Kyu Yoon, Min Hur, Hyeyeon Cho, Young Hyun Jeong, Ho-Jin Lee, Seong-Mi Yang, Won Ho Kim

**Affiliations:** 1 Department of Anesthesiology and Pain Medicine, Seoul National University Hospital, Seoul National University College of Medicine, 101 Daehak-ro, Jongno-gu, Seoul, 03080 Republic of Korea; 2 Department of Anesthesiology and Pain Medicine, School of Medicine, Ajou University, Suwon, Republic of Korea

**Keywords:** Medical research, Clinical trial design

## Abstract

We investigated whether two needle insertion techniques for ultrasound-guided internal jugular vein (IJV) catheterization differ in the number of needling attempts and complication rate between inexperienced and experienced practitioners. A total of 308 patients requiring IJV catheterization were randomly assigned into one of four groups: IJV catheterization performed by inexperienced practitioners using either Seldinger (IE-S; n = 78) or modified Seldinger technique (IE-MS; n = 76) or IJV catheterization performed by experienced practitioners using either Seldinger (E-S; n = 78) or modified Seldinger technique (E-MS; n = 76). All catheterizations were performed under the real-time ultrasound guidance. The number of needling attempts was not significantly different between the two techniques within each experience group (between IE-S vs. IE-MS *P* = 0.550, between E-S and E-MS *P* = 0.834). Time to successful catheterization was significantly shorter in the E-S group compared to E-MS group (*P* < 0.001) while no significant difference between IE-S and IE-MS groups (*P* = 0.226). Complication rate was not significantly different between the two techniques within each experience group. Practitioner’s experience did not significantly affect the clinical performance of needle insertion techniques during ultrasound-guided IJV catheterization except the time to successful catheterization. Regarding the number of needling attempts and complication rate, both techniques could be equally recommended regardless of practitioner’s experience.

Trial registration: clinicaltrials.gov (https://clinicaltrials.gov/ct2/show/NCT03077802).

## Introduction

Central venous catheterization (CVC) plays an important role for managements of the patients in the operating room or intensive care unit^1,2^. Internal jugular vein (IJV) catheterization is a preferred site due to better accessibility and safety than other central veins^3^. However, IJV catheterization is not free from mechanical complications such as venous hematoma^4–7^. Real-time ultrasound guidance during IJV catheterization significantly reduced these complication rates than anatomical landmark-based technique^8^, and a recent guideline recommended the use of ultrasound for CVC^9^.

Two different needle insertion techniques, including Seldinger and modified Seldinger techniques have been widely used to place a guidewire into the veins during CVC^10^. In the Seldinger technique (ST; thin-walled introducer needle technique), the desired vessel is punctured with a sharp and hollow introducer needle, and a guidewire is advanced through the needle. Meanwhile, the modified Seldinger technique (MST; catheter-over-needle technique) uses a needle covered with a guiding sheath. In the ST, the needle may migrate during guidewire insertion. However, MST provides relatively stable route into the vessel lumen by using a guiding sheath, but it can also cause problems such as kinking when a guiding sheath is advanced. Therefore, a recent guideline recommended that the needle insertion technique should be chosen by considering practitioner’s skill and experience^10^.

A previous study reported that ST showed favourable results than MST regarding the number of puncture attempts and the first-attempt success rate of needle and guidewire insertion during IJV catheterization^11^. However, in that study^11^, all procedures were performed only by practitioners who had more than 50 experiences of CVC. Their results did not provide us a meaningful information regarding the influence of practitioner’s experience on the clinical performance of two needle insertion techniques. Furthermore, to our knowledge, there has been no study comparing the effect of practitioner’s experience on the clinical performance of ST and MST during ultrasound-guided IJV catheterization.

We hypothesized that the clinical performance of two needle insertion techniques during ultrasound-guided IJV catheterization would be different depending on practitioner’s experience. In the present study, this hypothesis was evaluated by comparing the number of needling attempts until successful venous puncture and complication rates between ST and MST during IJV catheterization performed by inexperienced and experienced practitioners. From our results, we aimed to suggest ST or MST depending on practitioner’s experience. In addition, as a secondary goal, the effects of short-axis/out-of-plane and long-axis/in-plane ultrasound approaches on the clinical performance of two needle insertion techniques during IJV catheterization were evaluated by randomising the ST and MST into short- and long-axis approaches.

## Results

**Figure 1:**
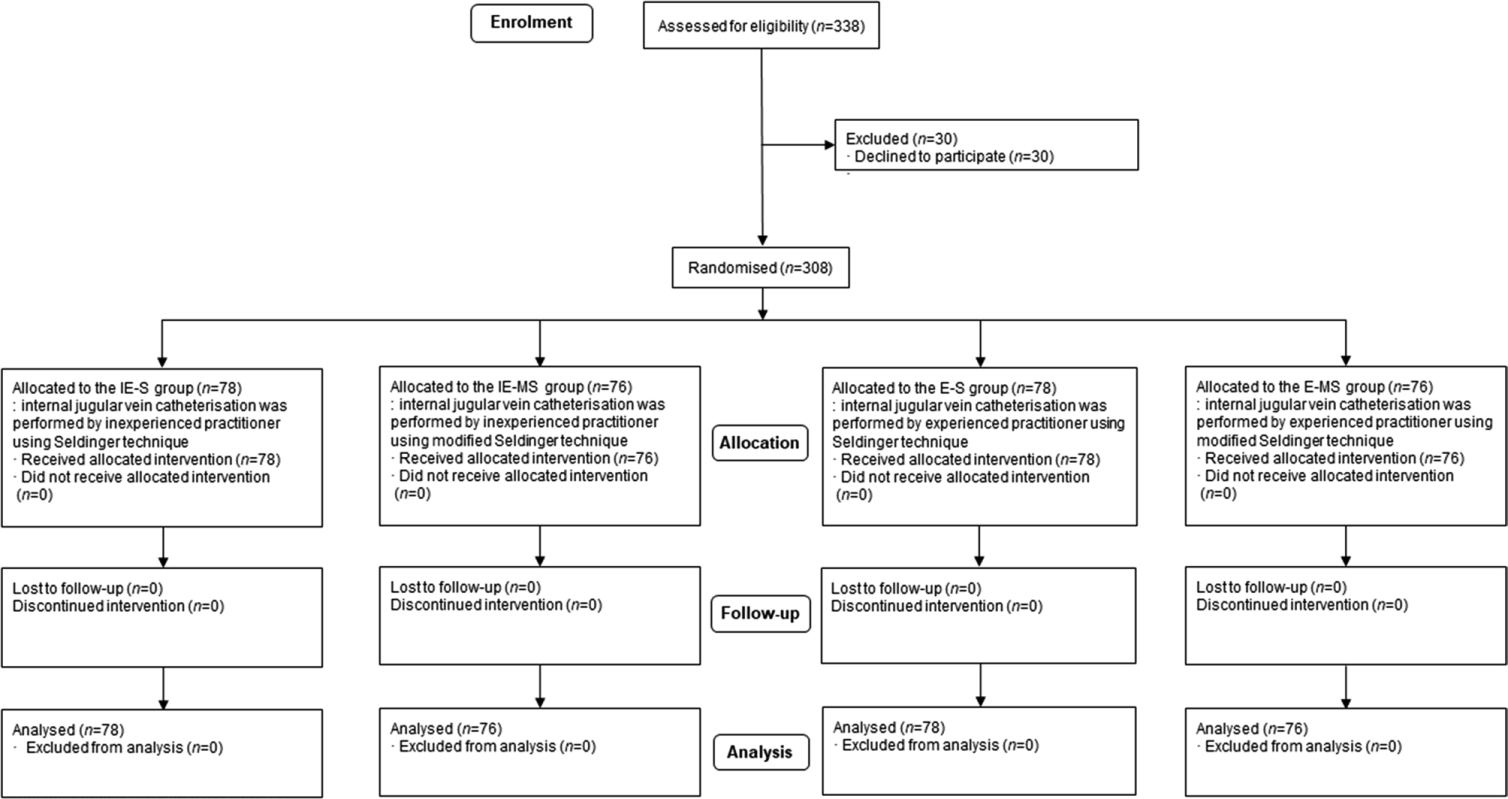
CONSORT flow diagram of the study.

Among 338 patients eligible for this study, thirty patients were excluded because they refused to participate in the study. The remaining 308 patients were randomly allocated to IE-S (n = 78), IE-MS (n = 76), E-S (n = 78), or E-MS (n = 76) group (Fig. 1), finished the study protocol, and were included in our final analysis. The baseline characteristics of the patients were compared among the four groups (Table 1).

**Table 1:** Comparison of baseline characteristics of patients between the four groups according to the practitioner’s experience and catheterization techniques (*n* = 308).

Characteristics	IE-S (*n* = 78)	IE-MS (*n* = 76)	E-S (*n* = 78)	E-MS (*n* = 76)
Age (years)	64 [54 to 70]	67 [58 to 74]	63 [53–72]	64 [56–70]
Male	50 (64.1)	49 (64.5)	59 (75.6)	49 (64.5)
Height (cm)	162.6 ± 7.6	163.3 ± 8.7	165.6 ± 8.1	163.0 ± 8.0
Weight (kg)	62.0 ± 10.7	65.0 ± 12.6	65.6 ± 11.4	63.8 ± 10.4
Body-mass index (kg m^-2^)	23.4 ± 3.2	24.3 ± 3.6	24.9 ± 3.2	24.0 ± 3.0
Internal jugular vein diameter* (mm)	18.2 ± 4.0	19.2 ± 4.2	17.8 ± 3.8	17.4 ± 4.1
Common carotid artery diameter (mm)	7.8 [7.1 to 8.6]	7.7 [6.8 to 8.6]	7.9 [6.9–8.8]	7.6 [6.7–8.4]
Percent overlap (%)	15.7 [7.1 to 25.7]	17.8 [12.9 to 27.7]	15.0 [6.6–21.0]	14.3 [5.4–22.9]
Coagulopathy	0 (0.0)	1 (1.3)	0 (0.0)	1 (1.3)

Values are presented as median [IQR], number (proportion) or mean ± SD.

Ultrasound-guided internal jugular vein catheterization was performed using either Seldinger technique or modified Seldinger technique by inexperienced practitioners in IE-S and IE-MS groups, respectively. Meanwhile, ultrasound-guided internal jugular vein catheterization was performed using either Seldinger technique or modified Seldinger technique by experienced practitioners in E-S and E-MS groups, respectively.

IE-S = inexperienced Seldinger technique, IE-MS = inexperienced modified Seldinger technique, E-S = experienced Seldinger technique, E-MS = experienced modified Seldinger technique. *There were two cases of missing data about vessel diameters in E-S and E-MS groups, respectively.

Ultrasound-guided IJV catheterization was successful in 300 patients (97.4%) and the eight patients with initial failure were successfully catheterized at the left IJV. The number of needing attempt until successful venous puncture as a continuous variable was not significantly different among the four group (IE-S 1 [1 to 1], IE-MS 1 [1 to 2], E-S 1 [1 to 1], E-MS 1 [1 to 1], *P* = 0.003) and between ST and MST in each experience group (IE-S vs. IE-MS *P* = 0.550; E-S vs. E-MS *P* = 0.834) (Table 2). The needling attempt as an incidence variable was not significantly different among the four groups (*P* < 0.007) and between two needle insertion techniques in each experience group (IE-S vs. IE-MS *P* = 0.379; E-S vs. E-MS *P* = 0.727) (Table 2).

**Table 2:** Comparison of catheterization-related study outcomes between the four groups according to the practitioner’s experience and catheterization techniques.

Characteristics	IE-S (*n* = 78)	IE-MS (*n* = 76)	E-S (*n* = 78)	E-MS (*n* = 76)	*P* values		
					Overall	IE-S versus IE-MS	E-S versus E-MS
Ultrasound guidance					0.567	0.428	0.914
Short-axis/out-of-plane	52 (66.7)	46 (60.5)	54 (69.2)	52 (68.4)			
Long-axis/in-plane	26 (33.3)	30 (39.5)	24 (30.8)	24 (31.6)			
Successful catheterization within three attempts	75 (96.2)	73 (96.1)	78 (100.0)	74 (97.4)	0.346	0.999	0.242
Needling attempts (n)	1 [1 to 1]	1 [1 to 1]	1 [1 to 1]	1 [1 to 1]	0.300	0.550	0.834
Needling attempts					0.007	0.379	0.727
1	58 (77.3)	54 (74.0)	71 (91.0)	68 (91.9)			
2	16 (21.3)	15 (20.5)	5 (6.4)	5 (6.8)			
3	1 (1.3)	4 (5.5)	2 (2.6)	1 (1.4)			
Guidewire insertion attempts					0.094	0.168	0.588
1	66 (88.0)	68 (93.2)	76 (97.4)	69 (93.2)			
2	5 (6.7)	4 (5.5)	1 (1.3)	4 (5.4)			
3	3 (4.0)	1 (1.4)	0 (0.0)	1 (1.4)			
4	1 (1.3)	0 (0.0)	1 (1.3)	0 (0.0)			
Catheter insertion attempts					0.858	0.206	0.235
1	74 (98.7)	69 (94.5)	78 (100.0)	72 (97.3)			
2	1 (1.3)	4 (5.5)	0 (0.0)	2 (2.7)			
Venous puncture type					0.072	0.949	0.442
Aspiration-on-advance	51 (68.0)	50 (68.5)	64 (82.1)	57 (77.0)			
Aspiration-on-withdrawal	24 (32.0)	23 (31.5)	14 (17.9)	17 (23.0)			
Dilation grade					< 0.001	0.004	< 0.001
I	55 (73.3)	34 (46.6)	68 (87.2)	35 (47.3)			
II	13 (17.3)	27 (37.0)	9 (11.5)	19 (25.7)			
III	7 (9.3)	12 (16.4)	1 (1.3)	20 (27.0)			
Time to successful catheterization (s)	170 [135 to 226]	183 [138 to 297]	101 [80 to 131]	130 [115 to 164]	< 0.001	0.226	< 0.001

Values are presented as number (proportion) or median [IQR].

For continuous variables, overall *P* values are the results of Kruskal–Wallis test and other *P* values are the results of Mann–Whitney *U* test between the designated two groups. For incidence variables, *P* values are the results of the chi-square test or Fisher’s exact test according to their expected counts.

IE-S = inexperienced Seldinger technique, IE-MS = inexperienced modified Seldinger technique, E-S = experienced Seldinger technique, E-MS = experienced modified Seldinger technique.

For secondary outcomes, the incidence of successful catheterization within three attempts, the number of guidewire and catheter insertion attempts, and the type of venous puncture did not differ significantly among the four groups or between needle insertion techniques in each experience group (Table 2). However, dilation grades were significantly lower in the groups using ST than those using MST regardless of practitioner’s experience (overall *P* < 0.001, IE-S vs. IE-MS *P* = 0.004; E-S vs. E-MS *P* < 0.001). For experienced practitioners, time to successful catheterization was significantly shorter in ST than MST (E-S 101 s [80 to 131] vs. E-MS 130 [115 to 164], *P* < 0.001) but this difference was not found for the catheterizations performed by inexperienced practitioners (IE-S 170 s [135 to 226] vs. IE-MS 183 [138 to 297], *P* = 0.226).

The incidences of overall catheterization-related complications were not significantly different between the four groups (*P* = 0.062) and did not differ within inexperienced (IE-S 15.4% vs. IE-MS 18.4%, *P* = 0.615) and experienced practitioners (E-S 2.6% vs. E-MS 10.5%, *P* = 0.055) (Table 3).

**Table 3:** Comparison of the catheterization-related complications between the four groups according to the practitioner’s experience and catheterization techniques.

Characteristics	IE-S (*n* = 78)	IE-MS (*n* = 76)	E-S (*n* = 78)	E-MS (*n* = 76)	*P* values		
					Overall	IE-S versus IE-MS	E-S versus E-MS
Overall complications	12 (15.4)	14 (18.4)	2 (2.6)	8 (10.5)	0.062	0.615	0.055
Arterial puncture	2 (2.6)	1 (1.3)	0 (0.0)	0 (0.0)	0.302	0.999	–
Venous hematoma	10 (12.8)	14 (18.4)	2 (2.6)	8 (10.5)	0.154	0.338	0.055
On ultrasound	9 (11.5)	13 (17.1)	2 (2.6)	8 (10.5)	0.257	0.324	0.055
Visible on skin	4 (5.1)	11 (14.5)	0 (0.0)	2 (2.6)	0.062	0.060	0.242
Pneumothorax	0 (0.0)	0 (0.0)	0 (0.0)	0 (0.0)	–	–	–
Hemothorax	0 (0.0)	0 (0.0)	0 (0.0)	0 (0.0)	–	–	–

Values are presented as number (proportion).

IE-S = inexperienced Seldinger technique, IE-MS = inexperienced modified Seldinger technique, E-S = experienced Seldinger technique, E-MS = experienced modified Seldinger technique.

*P* values are the results of the chi-square test or Fisher’s exact test according to their expected counts.

Baseline characteristics of the patients were compared again among the four groups according to the approaches of ultrasound guidance and needle insertion techniques (Supplementary Table S1). There were no significant differences between short- and long-axis approaches in any catheterization-related outcomes within each ST and MST groups (Supplementary Table S2). The catheterization-related complications were compared again among the four groups (Supplementary Table S3). When using MST, the incidences of venous hematoma were significantly higher in the short-axis approach than the long-axis approach (MS-SA 21.4% vs. MS-LA 1.9%, *P* = 0.001), while it was comparable between the groups when using ST.

In the comparison of the inexperienced and experienced practitioners regardless of needle insertion techniques (Supplementary Table S4), there were significant difference in number of needling attempts, the type of venous puncture, time to successful catheterization, and incidence of venous hematoma, all favouring the experienced group.

In the comparisons of ST and MST regardless of experience (Supplementary Table S5), ST group showed favourable profiles regarding dilation grades and time to successful catheterization than MST group. In the comparison of short- and long-axis approaches (Supplementary Table S6), short-axis guidance resulted in significantly more mechanical complications than long-axis guidance (short-axis 16.2% vs. long-axis 2.9%, *P* < 0.001).

## Discussion

In this study, our primary outcome of the number of needling attempts until successful venous puncture was not significantly different between ST and MST regardless of practitioner’s experience. The incidence of mechanical complications of two needle insertion techniques during IJV catheterization were also similar between ST and MST in both inexperienced and experienced groups, suggesting that practitioner’s experience does not influence the success and complication rates whether ST or MST is used. However, time to successful catheterization was significantly shorter in ST than MST in experienced groups while no difference was observed in inexperienced groups. This was the only difference depending on the degree of experience. Dilation grade was significantly better for ST than MST regardless of the experience level. In our post-hoc analysis regarding approaches of ultrasound guidance, long-axis approach was associated with significantly less incidence of venous hematoma. As expected, overall comparison between inexperienced and experienced groups revealed that experienced groups showed significantly better profiles of catheterization-related outcomes and complication rates. Our results suggest that ST or MST could be equally recommended to both inexperienced and experienced groups.

To our knowledge, this is the first study to investigate the effects of practitioner’s experience on the clinical performance of ST and MST during ultrasound-guided IJV catheterization. Previous studies which compared ST and MST showed inconsistent results depending on the patient population or target vessel^11–14^, suggesting further well-designed trials are still required. For adult patients, ST showed higher success rate of guidewire insertion at the first-attempt during IJV catheterization^11^ and lower complication rate during subclavian vein catheterization than MST^12^. Meanwhile, there were no significant differences in the success rate of puncture and guidewire insertion at the first-attempt between two needle insertion techniques in pediatric patients^13^. Contrary to the adult patients, MST was superior to ST regarding the success rate of guidewire insertion at the first-attempt for IJV catheterization of neonate^14^. However, all abovementioned studies did not consider the effect of practitioner’s experience on clinical outcomes and the varying degree of experience could variably affect the study results.

Practitioner’s experience was reported to play an important role in the success rate of IJV catheterization^15,16^. We compared the IJV catheterization-related outcomes between ST and MST within two groups with different level of experience for IJV catheterization. Our hypothesis was that ST and MST may have different profiles on catheterization-related parameters and complications between inexperienced and experienced groups and that different needle insertion technique could be suggested according to the practitioner’s experience. However, we could not find any significant difference between ST and MST in each experience groups except the time to successful catheterization. For experienced practitioners, it took less time to catheterize successfully with ST than MST. There have been only few studies comparing ST and MST for IJV cannulation^11^. A previous randomized trial reported that ST is superior to MST regarding the success rate of needle and guidewire insertion at the first attempt^11^. However, this study did not compare the time to successful catheterization. We also found that dilation grade was better in ST than MST regardless of experience level. Therefore, we could suggest ST rather than MST regarding only the dilation grade regardless of the degree of experience and suggest ST rather than MST regarding only the time to successful catheterization for experienced practitioners. In terms of mechanical complications, however, there were no significant differences between the four groups and between inexperience and experienced practitioners. Current guidelines also recommend that practitioners can choose either ST or MST by their skills and experience^10,17^. In line with these guidelines, we could suggest that inexperienced practitioners may choose either technique without significant complications similarly as experienced practitioners. Safe procedure is possible for both techniques even by inexperience practitioners.

However, our results should be interpreted cautiously due to the following possible biases. Firstly, the degree of experience may vary within inexperienced or experienced groups and criteria to define experienced practitioner could be inaccurate. We defined experienced practitioners as those who had experience of more than 50 CVCs according to a previous study^11^. However, according to prior investigations, skills of ultrasound-guided IJV catheterization can be easily acquired with training less than 10 cases^18^, and practitioners with experiences less than 25 CVCs caused significantly more complications than those with more than 25 CVC experiences^19^. Therefore, experienced practitioners may have been assigned to inexperienced groups. However, we validated the practitioner’s experience group assignment before enrolment and all the practitioners in the inexperienced groups were first-year junior residents with less than 10 CVC experiences. Also, a practitioner in the inexperienced groups was additionally excluded from the inexperienced groups when he or she gained experience of more than 25 CVCs. Secondly, the presence of staff anesthesiologists with inexperienced practitioners during catheterization may have affected our results, although our protocol prevented any intervention by the staff. A previous study of infants failed to demonstrate the effects of practitioner’s experience on the success rate of IJV catheterization^20^, which was suggested to be due to the effect of supervising during catheterization^21^.

Ultrasound-guided CVC can be performed in short- or long-axis approach depending on the orientation of ultrasound probe to the vessel. Short-axis/out-of-plane approach could visualize relative spatial relationship of vessels better than long-axis/in-plane approach^22^ and provide a higher first-attempt success rate of IJV catheterization performed by experienced anesthesiologists^23^. Meanwhile, long-axis/in-plane approach could provide the vision of entire needle pathway and depth of the needle tip during needle insertion for venous puncture. This could reduce the incidence of posterior wall puncture and venous hematoma^24^. A recent meta-analysis reported that there were no significant differences in the incidence of arterial puncture, total and first-pass success rate, and number of needling attempts between short- and long-axis approaches^25^. In our results, the approach of ultrasound guidance did not significantly affect needling attempts until successful venous puncture or the success rate of catheterization between needle insertion techniques. However, short-axis/out-of-plane approach caused significantly higher incidence of venous hematoma than long-axis/in-plane approach regardless of needle insertion techniques.

This study also had several limitations. Firstly, practitioners and outcome assessor could not be blinded to the group assignment, which may affect our results. However, they did not know the purpose of our study and data analyser was blinded to the group assignment. Secondly, we defined experienced practitioners as those who have more than 50 experiences of CVCs. However, a prior investigation reported that ultrasound-guided IJV catheterization can be easily acquired with training less than 10 cases^18^. Therefore, practitioners included in the inexperienced groups could not represent lack of experience of CVCs. Thirdly, randomization of approaches of ultrasound guidance, i.e. short- vs. long-axis approach was performed only after enrolment of about 100 patients. Therefore, the analysis results regarding approaches of ultrasound guidance were from incompletely-randomized trial.

In conclusions, practitioner’s experience did not significantly influence the needling attempts until successful venous puncture during ultrasound-guided IJV catheterization. Other catheterization-related outcomes and complications did not differ between needle insertion techniques according to the practitioner’s experience except the time to successful catheterization. Time to successful catheterization was significantly shorter in ST than MST only in the experienced groups. Dilation grade was significantly better in ST compared to MST regardless of the degree of experience. However, given no difference in our primary outcome and most of the secondary catheterization-related outcomes and complications, ST or MST could be equally recommended to both inexperienced and experienced practitioners. Even inexperienced practitioners may choose either technique without significant complications similarly as experienced practitioners.

## Methods

After the Institutional Review Board (IRB) of Seoul National University Hospital approved this randomized controlled trial (1506–126-684), the study protocol was registered at the ClinicalTrials.gov (NCT03077802; data of registration 13/03/2017). This study was performed in accordance with Good Clinical Practice Guidelines and stuck to the applicable Consolidated Standards of Reporting Trials (CONSORT) guidelines. Written informed consent was obtained from all patients before enrolment. Adult patients who were scheduled for elective surgery and who required IJV catheterization between March 2017 and December 2018 at our institution were enrolled in this study. Patients who refused to participate in the study, had current infection around skin puncture site, diaphragm dysfunction in contralateral side, anatomic anomaly of neck vessels, previous history of neck surgery, and recent (less than 1 month) history of right IJV catheterization were excluded. Eligibility was assessed and patients were enrolled by an investigator (M.H.).

Blocked randomization was performed with a randomly selected block size of four. Random allocation sequence was generated by internet-based computer program by an investigator (M.H.). All patients were randomly allocated to one of the following four groups; IJV catheterization performed by inexperienced practitioners using either Seldinger (IE-S) or modified Seldinger technique (IE-MS) or IJV catheterization performed by experienced practitioners using either Seldinger (E-S) or modified Seldinger technique (E-MS). Inexperienced practitioners were defined as junior residents who had experience of less than 50 CVCs according to a previous study^11^, and experienced practitioners were defined as staff anesthesiologists who had experience of more than 50 CVCs in both techniques. All practitioners who participated in the present study learned the ultrasound-guided central venous catheterization through on-the-job training. In addition to the number of experience of CVCs, the practitioners were initially further validated by demonstrating their practice of CVC with ST or MST in front of two investigators. The two investigators evaluated their skill of CVC and concluded whether practitioners could be classified as inexperienced or experienced. All catheterization procedures were performed by 8 junior residents and 4 staff anesthesiologists. More number of inexperienced practitioners was required because some of them gained experience during the study period. If an inexperienced practitioner gained experience more than 25 CVCs, the practitioners stopped to participate in our trial. This was to consider the results of a previous study using different cutoff for experience^19^. When junior residents performed CVC, a supervising staff anesthesiologist attended but did not interrupt their practice until success or failure of the catheterization was determined. Patients, surgeons, and data analysers were blinded to this study and group assignment. The allocation order was concealed in an opaque envelope and was disclosed just before performing IJV catheterization by an investigator (W.K.). When 100 patients finished the study protocol and 208 patients remained until full enrolment, randomization regarding the approach of ultrasound guidance including short-axis/out-of-plane and long-axis/in-plane approaches was added to the study protocol after approval of the IRB. This was to evaluate whether the clinical performance of two needle insertion techniques would be different depending on the approach of ultrasound guidance.

Following induction of anesthesia, patients were placed in the supine and Trendelenburg position. At the level of cricoid cartilage, diameters and the extent of overlapping of right IJV and common carotid artery were measured by using ultrasound (Vivid-q; GE Healthcare, Wauwatosa, WI, USA), and skin puncture was also performed at this level. Using real-time ultrasound guidance, a 7-Fr double lumen central venous catheter (Arrow International Inc., Reading, PA, USA) was placed via either ST or MST. An 18-gauge introducer needle in the groups using ST and catheter-over-needle in the groups using MST were used for venous puncture, respectively. During venous puncture, needles were directed with an angle of 30° from the skin with bevel-up needle tip position. Successful venous puncture was confirmed by free-flow venous blood return within the syringe and obtaining venous pressure waveform on the monitor. In case of arterial puncture, the needle was removed, and manual compression was applied for more than 5 min. After successful venous puncture, a guidewire was placed through the introducer or catheter-over needle. We also recorded whether venous puncture was achieved during needle advance or withdrawal as the type of venous puncture. The number of needling attempts for successful venous puncture, and the number of guidewire and catheter insertion attempts were counted. We also evaluated the dilation grade, which was defined as difficulty of tissue dilation (I: easy to dilate, II: difficult to dilate, III: difficult to dilate, even required a scalpel incision)^11^. We measured time to successful catheterization as the time interval from skin puncture to catheter insertion. If each step of catheterization did not succeed within three needling attempts, practitioners were changed for patient’s safety, and if another practitioner also failed to catheterize within three attempts, this case was recorded as the failure in catheterization. If right IJV catheterization did not succeed, left IJV or subclavian vein was catheterized instead.

Regarding mechanical complications, arterial puncture and hematoma formation were evaluated by using ultrasound at three different time points (before skin puncture, after guidewire insertion, and after catheter placement). Pneumothorax and hemothorax were evaluated by using ultrasound or chest radiography after completion of catheterization. All these catheterization-related parameters and mechanical complications were measured and recorded by a staff anesthesiologist who was not involved in our study and did not know the purpose of our study.

The primary outcome measure was the number of needling attempts until successful venous puncture. Secondary outcomes were the number of guidewire and catheter insertion attempts, dilation grades, type of venous puncture, and time to successful catheterization, and incidence of mechanical complications including arterial puncture, venous hematoma, pneumothorax, and hemothorax.

According to a previous study^11^, mean ± SD numbers of needling attempts for successful venous puncture among 134 patients with IJV catheterization were 1.1 ± 0.4 and 1.3 ± 0.6 in groups using ST and MST, respectively. For our sample size, we hypothesized that there is a significant difference in the number of needling attempts between ST and MST groups in the experienced group as the previous study^11^ and there is no significant difference in the inexperienced group. For the experienced group, 138 patients per group were required with a type I error of 0.05 and a power of 90%. Considering a possible dropout rate of 10%, a total of 308 patients were required for enrolment. For the inexperienced group, 122 patients per group were required with the same type error and power for non-inferiority between groups. For this calculation, means ± SD of the number of needling attempts for successful puncture were 1.4 ± 0.4 for both groups and non-inferiority margin was 0.15.

### Statistical analysis

Data were expressed as number (proportion), mean ± SD, and median [IQR]. Continuous variables were compared using Kruskal–Wallis test among four groups and Mann–Whitney U test between any two groups. Discrete variables were compared using the chi-squared test or Fisher’s exact test depending on the expected count. All statistical analyses were performed using SPSS software (version 25.0; IBM Corp., Armonk, NY, USA). A *P* value < 0.002 (0.05/30 = 0.002) was considered to indicate a statistical significance after the Bonferroni correction to adjust for three comparisons per each outcome and number of outcomes of ten.

Firstly, as the primary analysis, the primary and secondary outcomes were compared among the four study groups (IE-S, IE-MS, E-S, and E-MS). Then, the outcome variables were compared between ST and MST within each experience group to evaluate whether our study outcomes differ between two needle insertion techniques depending on the practitioner’s experience. Secondly, as a post-hoc analysis, to evaluate the effect of short- and long-axis approaches on the outcome variable, we divided our patients into four groups (S-SA; ST with short-axis approach, S-LA; ST with long-axis approach, MS-SA; MST with short-axis approach, and MS-LA; MST with long-axis approach) and compared the study outcomes. Thirdly, as a post-hoc analysis, we divided our patients into two groups according to the degree of experience, type of needle insertion techniques, and approaches of ultrasound guidance, respectively and compared study outcomes between the two groups. This was to evaluate the effect of experience, needle insertion techniques, and approaches of ultrasound guidance on our study outcomes regardless of other factors.

## Supplementary Information

Supplementary Information
