# Supplementary material for: Effects of practitioner’s experience on the clinical performance of ultrasound-guided central venous catheterization: a randomized trial
Source: Sci Rep. 2021 Mar 24;11:6726. doi: 10.1038/s41598-021-86322-y (PMC7991409; doi:10.1038/s41598-021-86322-y)
Supplement: Supplementary file 1 — Supplementary Information [file 41598_2021_86322_MOESM1_ESM.docx]

**Effects of practitioner’s experience on the clinical performance of ultrasound-guided central venous catheterization: a randomized trial**

**Supplemental Materials**

Hyun-Kyu Yoon ^1*^, Min Hur ^2*^, Hyeyeon Cho^1^, Young Hyun Jeong^1^, Ho-Jin Lee ^1^, Seong-Mi Yang ^1^, and Won Ho Kim ^1^

^1^ Department of Anesthesiology and Pain Medicine, Seoul National University Hospital, Seoul National University College of Medicine, Seoul, Republic of Korea

^2^ Department of Anesthesiology and Pain Medicine, Ajou University, School of Medicine, Suwon, Republic of Korea

**List of Supplemental Materials**

**Table S1** Baseline characteristics of patients between the four groups according to catheterization technique and axial approach (*n*=308).

**Table S2** Comparison of catheterization-related parameters between the four groups according to catheterization technique and axial approach.

**Table S3** Comparison of catheterization-related complications between the four groups according to catheterization technique and axial approach (*n*=308).

**Table S4** Comparison of catheterization-related outcomes and complications between inexperienced and experienced practitioner.

**Table S5** Comparison of catheterization-related outcomes and complications between Seldinger and modified Seldinger technique.

**Table S6** Comparison of catheterization-related outcomes and complications between short-axis and long-axis approaches

**Table S1** Baseline characteristics of patients between the four groups according to catheterization techniques and axial approaches (*n*=308).

| **Characteristics** | **S-SA**  **(*n*=106)** | **S-LA**  **(*n*=50)** | **MS-SA**  **(*n*=98)** | **MS-LA**  **(*n*=54)** |
| --- | --- | --- | --- | --- |
| Age (years) | 63 [52 to 71] | 65 [58 to 71] | 64 [56 to 72] | 65 [58 to 72] |
| Male | 77 (72.6) | 32 (64.0) | 61 (62.2) | 37 (68.5) |
| Height (cm) | 164.8 ± 8.1 | 162.5 ± 7.7 | 162.7 ± 8.5 | 163.9 ± 8.1 |
| Weight (kg) | 64.3 ± 11.2 | 62.8 ± 11.0 | 63.9 ± 11.0 | 65.3 ± 12.5 |
| Body-mass index (kg m^-2^) | 23.6 ± 3.3 | 23.7 ± 3.1 | 24.1 ± 3.3 | 24.2 ± 3.4 |
| Internal jugular vein diameter^*^ (mm) | 18.2 ± 4.1 | 17.5 ± 3.3 | 18.0 ± 3.6 | 19.0 ± 5.2 |
| Common carotid artery diameter (mm) | 7.9 [7.1 to 8.8] | 7.8 [6.8 to 8.3] | 7.5 [6.7 to 8.5] | 7.8 [6.9 to 8.9] |
| Percent overlap (%) | 15.2 [7.9 to 23.4] | 15.4 [4.7 to 24.4] | 15.1 [9.1 to 23.0] | 17.3 [7.7 to 27.6] |
| Coagulopathy | 0 (0.0) | 0 (0.0) | 2 (2.0) | 0 (0.0) |

Values are presented as median [IQR], number (proportion), or mean ± SD.

Ultrasound-guided internal jugular vein catheterization was performed using Seldinger technique under either short-axis/out-of-plane or long-axis/in-plane approach in S-SA and S-LA groups, respectively. Meanwhile, ultrasound-guided internal jugular vein catheterization was performed using modified Seldinger technique under either short-axis/out-of-plane or long-axis/in-plane approach in MS-SA and MS-LA groups, respectively.

S-SA = Seldinger technique with short-axis approach, S-LA = Seldinger technique with long-axis approach, MS-SA = modified Seldinger technique with short-axis approach, MS-LA = modified Seldinger technique with long-axis approach.

^*^There were two cases of missing data about vessel diameters in S-SA and MS-SA groups, respectively.

**Table S2** Comparison of catheterization-related parameters between the four groups according to catheterization techniques and axial approaches.

| **Characteristics** | **S-SA**  **(*n*=106)** | **S-LA**  **(*n*=50)** | **MS-SA**  **(*n*=98)** | **MS-LA**  **(*n*=54)** | ***P* values** | | |
| --- | --- | --- | --- | --- | --- | --- | --- |
|  |  |  |  |  | **Overall** | **S-SA vs. S-LA** | **MS-SA vs. MS-LA** |
| Practitioners |  |  |  |  | 0.685 | 0.732 | 0.309 |
| Inexperienced | 52 (49.1) | 26 (52.0) | 46 (46.9) | 30 (55.6) |  |  |  |
| Experienced | 54 (50.9) | 24 (48.0) | 52 (53.1) | 24 (44.4) |  |  |  |
| Successful catheterization within three attempts^*^ | 104 (98.1) | 49 (98.0) | 93 (94.9) | 54 (100.0) | 0.898 | 0.999 | 0.161 |
| Needling attempts (n) | 1 [1 to 1] | 1 [1 to 1] | 1 [1 to 1] | 1 [1 to 1] | 0.930 | 0.590 | 0.825 |
| Needling attempts |  |  |  |  | 0.773 | 0.887 | 0.473 |
| 1 | 89 (85.6) | 40 (81.6) | 77 (82.8) | 45 (83.3) |  |  |  |
| 2 | 12 (11.5) | 9 (18.4) | 11 (11.8) | 9 (16.7) |  |  |  |
| 3 | 3 (2.9) | 0 (0.0) | 5 (5.4) | 0 (0.0) |  |  |  |
| Guidewire insertion attempts |  |  |  |  | 0.471 | 0.779 | 0.752 |
| 1 | 96 (92.3) | 46 (93.9) | 88 (94.6) | 49 (90.7) |  |  |  |
| 2 | 4 (3.8) | 2 (4.1) | 3 (3.2) | 5 (9.3) |  |  |  |
| 3 | 3 (2.9) | 0 (0.0) | 2 (2.2) | 0 (0.0) |  |  |  |
| 4 | 1 (1.0) | 1 (2.0) | 0 (0.0) | 0 (0.0) |  |  |  |
| Catheter insertion attempts |  |  |  |  | 0.009 | 0.320 | 0.193 |
| 1 | 104 (100.0) | 48 (98.0) | 91 (97.8) | 50 (92.6) |  |  |  |
| 2 | 0 (0.0) | 1 (2.0) | 2 (2.2) | 4 (7.4) |  |  |  |
| Venous puncture type |  |  |  |  | 0.980 | 0.739 | 0.071 |
| Aspiration-on-advance | 79 (76.0) | 36 (73.5) | 63 (67.7) | 44 (81.5) |  |  |  |
| Aspiration-on-withdrawal | 25 (24.0) | 13 (26.5) | 30 (32.3) | 10 (18.5) |  |  |  |
| Dilation grade |  |  |  |  | <0.001 | 0.310 | 0.731 |
| Ⅰ | 80 (76.9) | 43 (87.8) | 44 (47.3) | 25 (46.3) |  |  |  |
| Ⅱ | 19 (18.3) | 3 (6.1) | 30 (32.3) | 16 (29.6) |  |  |  |
| Ⅲ | 5 (4.8) | 3 (6.1) | 19 (20.4) | 13 (24.1) |  |  |  |
| Time to successful catheterization (s) | 133 [100 to 177] | 126 [93 to 191] | 151 [123 to 201] | 153 [114 to 263] | 0.003 | 0.851 | 0.920 |

Values are presented as number (proportion) or median [IQR].

For continuous variables, overall *P* values are the results of Kruskal-Wallis test and other *P* values are the results of Mann-Whitney U test between the designated two groups. For incidence variables, *P* values are the results of the chi-square test or Fisher’s exact test according to their expected counts.

S-SA = Seldinger technique with short-axis approach, S-LA = Seldinger technique with long-axis approach, MS-SA = modified Seldinger technique with short-axis approach, MS-LA = modified Seldinger technique with long-axis approach.

^*^Internal jugular vein catheterization was not successful within three attempts in eight patients.

**Table S3.** Comparison of catheterization-related complications between the four groups according to catheterization techniques and axial approaches (*n*=308).

| **Characteristics** | **S-SA**  **(*n*=106)** | **S-LA**  **(*n*=50)** | **MS-SA**  **(*n*=98)** | **MS-LA**  **(*n*=54)** | ***P* values** | | |
| --- | --- | --- | --- | --- | --- | --- | --- |
|  |  |  |  |  | **Overall** | **S-SA vs.**  **S-LA** | **MS-SA vs. MS-LA** |
| Total complications | 12 (11.3) | 2 (4.0) | 21 (21.4) | 1 (1.9) | 0.913 | 0.228 | 0.001 |
| Arterial puncture | 0 (0.0) | 2 (4.0) | 1 (1.0) | 0 (0.0) | 0.097 | 0.101 | 0.999 |
| Venous hematoma | 12 (11.3) | 0 (0.0) | 21 (21.4) | 1 (1.9) | 0.995 | 0.010 | 0.001 |
| on ultrasound | 11 (10.4) | 0 (0.0) | 20 (20.4) | 1 (1.9) | 0.919 | 0.017 | 0.002 |
| visible on skin | 4 (3.8) | 0 (0.0) | 13 (13.3) | 0 (0.0) | 0.440 | 0.306 | 0.004 |
| Pneumothorax | 0 (0.0) | 0 (0.0) | 0 (0.0) | 0 (0.0) | - | - | - |
| Hemothorax | 0 (0.0) | 0 (0.0) | 0 (0.0) | 0 (0.0) | - | - | - |

Values are presented as number (proportion).

*P* values are the results of the chi-square test or Fisher’s exact test according to their expected counts.

S-SA = Seldinger technique with short-axis approach, S-LA = Seldinger technique with long-axis approach, MS-SA = modified Seldinger technique with short-axis approach, MS-LA = modified Seldinger technique with long-axis approach.

**Table S4** Comparison of catheterization-related outcomes and complications between inexperienced and experienced practitioners.

| **Characteristics** | **Inexperienced**  **(*n*=154)** | **Experienced**  **(*n*=154)** | **Relative risk**  **or mean difference (95% CI)** | ***P* value** |
| --- | --- | --- | --- | --- |
| Ultrasound guidance |  |  |  | 0.335 |
| Short-axis/out-of-plane | 98 (63.6) | 106 (68.8) | - |  |
| Long-axis/in-plane | 56 (36.4) | 48 (31.2) | - |  |
| Successful catheterization within three  attempts (*n*=308) | 148 (96.1) | 152 (98.7) | 0.33 (0.06 to 1.12) | 0.283 |
| Needling attempts (n) (*n*=300) | 1 [1 to 1] | 1 [1 to 1] | 0.17 (0.07 to 0.27) | <0.001 |
| Needling attempts |  |  |  | <0.001 |
| 1 | 112 (75.7) | 139 (91.4) | 0.29 (0.15 to 0.58) |  |
| 2 | 31 (20.9) | 10 (6.6) | 3.76 (1.77 to 7.99) |  |
| 3 | 5 (3.4) | 3 (2.0) | 1.74 (0.41 to 7.40) |  |
| Guidewire insertion attempts |  |  |  | 0.138 |
| 1 | 134 (90.5) | 145 (95.4) | 0.46 (0.18 to 1.18) |  |
| 2 | 9 (6.1) | 5 (3.3) | 1.90 (0.62 to 5.82) |  |
| 3 | 4 (2.7) | 1 (0.7) | 4.19 (0.46 to 38.0) |  |
| 4 | 1 (0.7) | 1 (0.7) | 1.03 (0.06 to 16.6) |  |
| Catheter insertion attempts |  |  |  | 0.278 |
| 1 | 143 (96.6) | 150 (98.7) | 0.38 (0.07 to 2.00) |  |
| 2 | 5 (3.4) | 2 (1.3) | 2.62 (0.50 to 13.7) |  |
| Venous puncture type (*n*=300) |  |  |  | 0.025 |
| Aspiration-on-advance | 101 (68.2) | 121 (79.6) | 0.55 (0.33 to 0.93) |  |
| Aspiration-on-withdrawal | 47 (31.8) | 31 (20.4) | 1.82 (1.08 to 3.07) |  |
| Dilation grade (*n*=300) |  |  |  | 0.424 |
| Ⅰ | 89 (60.1) | 103 (67.8) | 0.72 (0.45 to 1.15) |  |
| Ⅱ | 40 (27.0) | 28 (18.4) | 1.64 (0.95 to 2.84) |  |
| Ⅲ | 19 (12.8) | 21 (13.8) | 0.92 (0.47 to 1.79) |  |
| Time to successful catheterization (s) (*n*=300) | 179 [135 to 256] | 122 [95 to 148] | 12.6 (61.4 to 110.9) | <0.001 |
| Total complications (*n*=308) | 26 (16.9) | 10 (6.5) | 2.93 (1.36 to 6.30) | 0.005 |
| Arterial puncture | 3 (1.9) | 0 (0.0) | - | 0.248 |
| Venous hematoma | 24 (15.6) | 10 (6.5) | 2.66 (1.23 to 5.77) | 0.011 |
| on ultrasound | 22 (14.3) | 10 (6.5) | 2.40 (1.10 to 5.26) | 0.025 |
| Visible on skin | 15 (9.7) | 2 (1.3) | 8.20 (1.84 to 36.51) | 0.001 |
| Pneumothorax | 0 (0.0) | 0 (0.0) | - | - |
| Hemothorax | 0 (0.0) | 0 (0.0) | - | - |

Values are presented as number (proportion) or median [IQR].

*P* values are the results of Mann-Whitney U test for continuous variable or the chi-square test or Fisher’s exact test according to their expected counts.

**Table S5** Comparison of catheterization-related outcomes and complications between Seldinger and modified Seldinger techniques.

| **Characteristics** | **Seldinger technique**  **(*n*=156)** | **Modified Seldinger technique**  **(*n*=152)** | **Relative risk or mean difference (95% CI)** | ***P* value** |
| --- | --- | --- | --- | --- |
| Ultrasound-guidance |  |  |  | 0.519 |
| Short-axis/out-of-plane | 106 (67.9) | 98 (64.5) | - |  |
| Long-axis/in-plane | 50 (32.1) | 54 (35.5) | - |  |
| Successful catheterization within three attempts (*n*=308) | 153 (98.1) | 147 (96.7) | 1.74 (0.41 to 7.40) | 0.497 |
| Needling attempts (n) (*n*=300) | 1 [1 to 1] | 1 [1 to 1] | -0.03 (-0.07 to 0.01) | 0.723 |
| Needling attempts |  |  |  | 0.600 |
| 1 | 129 (84.3) | 122 (83.0) | 0.94 (0.39 to 2.29) |  |
| 2 | 21 (13.7) | 20 (13.6) | 0.71 (0.24 to 2.10) |  |
| 3 | 3 (2.0) | 5 (3.4) | 1.45 (0.24 to 8.80) |  |
| Guidewire insertion attempts |  |  |  | 0.441 |
| 1 | 142 (92.8) | 137 (93.2) | 0.94 (0.39 to 2.29) |  |
| 2 | 6 (3.9) | 8 (5.4) | 0.71 (0.24 to 2.10) |  |
| 3 | 3 (2.0) | 2 (1.4) | 1.45 (0.24 to 8.80) |  |
| 4 | 2 (1.3) | 0 (0.0) | - |  |
| Catheter insertion attempts |  |  |  | 0.062 |
| 1 | 152 (99.3) | 141 (95.9) | 6.47 (0.77 to 54.4) |  |
| 2 | 1 (0.7) | 6 (4.1) | 0.16 (0.02 to 1.30) |  |
| Venous puncture type (*n*=300) |  |  |  | 0.639 |
| Aspiration-on-advance | 115 (75.2) | 107 (72.8) | 1.31 (0.68 to 1.90) |  |
| Aspiration-on-withdrawal | 38 (24.8) | 40 (27.2) | 0.88 (0.53 to 1.48) |  |
| Dilation grade (*n*=300) |  |  |  | <0.001 |
| Ⅰ | 123 (80.4) | 69 (46.9) | 4.64 (2.77 to 7.75) |  |
| Ⅱ | 22 (14.4) | 46 (31.3) | 0.37 (0.21 to 0.65) |  |
| Ⅲ | 8 (5.2) | 32 (21.8) | 0.20 (0.09 to 0.45) |  |
| Time to successful catheterization (s) (*n*=300) | 131 [97 to 186] | 151 [121 to 217] | -25.2 (-52.6 to -2.1) | <0.001 |
| Total complications (*n*=308) | 14 (9.0) | 22 (14.5) | 0.58 (0.29 to 1.19) | 0.133 |
| Arterial puncture | 2 (1.3) | 1 (0.7) | 1.96 (0.18 to 21.9) | 0.999 |
| Venous hematoma | 12 (7.7) | 22 (14.5) | 0.49 (0.23 to 1.03) | 0.058 |
| on ultrasound | 11 (7.1) | 21 (13.8) | 0.47 (0.22 to 1.02) | 0.052 |
| Visible on skin | 4 (2.6) | 13 (8.6) | 0.28 (0.09 to 0.88) | 0.021 |
| Pneumothorax | 0 (0.0) | 0 (0.0) | - | - |
| Hemothorax | 0 (0.0) | 0 (0.0) | - | - |

Values are presented as number (proportion) or median [IQR].

*P* values are the results of Mann-Whitney U test for continuous variable or the chi-square test or Fisher’s exact test according to their expected counts.

**Table S6** Comparison of catheterization-related outcomes and complications between short-axis and long-axis approaches.

| **Characteristics** | **Short-axis approach**  **(*n*=204)** | **Long-axis approach**  **(*n*=104)** | **Relative risk**  **(95% CI)** | ***P* value** |
| --- | --- | --- | --- | --- |
| Practitioners |  |  |  | 0.335 |
| Inexperienced | 98 (48.0) | 56 (53.8) | - |  |
| Experienced | 106 (52.0) | 48 (46.2) | - |  |
| Successful catheterization within three attempts (*n*=308) | 197 (96.6) | 103 (99.0) | 0.27 (0.03 to 2.25) | 0.274 |
| Needling attempts (n) (*n*=300) | 1 [1 to 1] | 1 [1 to 1] | 0.02 (-0.09 to 0.13) |  |
| Needling attempts |  |  |  | 0.675 |
| 1 | 166 (84.3) | 85 (82.5) | 1.13 (0.60 to 2.14) |  |
| 2 | 23 (11.7) | 18 (17.5) | 0.62 (0.32 to 1.22) |  |
| 3 | 8 (4.1) | 0 (0.0) | - |  |
| Guidewire insertion attempts |  |  |  | 0.928 |
| 1 | 184 (93.4) | 95 (92.2) | 1.19 (0.48 to 2.98) |  |
| 2 | 7 (3.6) | 7 (6.8) | 0.51 (0.17 to 1.48) |  |
| 3 | 5 (2.5) | 0 (0.0) | - |  |
| 4 | 1 (0.5) | 1 (1.0) | 0.52 (0.03 to 8.41) |  |
| Catheter insertion attempts |  |  |  | 0.049 |
| 1 | 195 (99.0) | 98 (95.1) | 4.97 (1.05 to 26.1) |  |
| 2 | 2 (1.0) | 5 (4.9) | 0.20 (0.04 to 1.06) |  |
| Venous puncture type (*n*=300) |  |  |  | 0.295 |
| Aspiration-on-advance | 142 (72.1) | 80 (77.7) | 0.74 (0.43 to 1.30) |  |
| Aspiration-on-withdrawal | 55 (27.9) | 23 (22.3) | 1.35 (0.77 to 2.36) |  |
| Dilation grade (*n*=300) |  |  |  | 0.975 |
| Ⅰ | 124 (62.9) | 68 (66.0) | 0.87 (0.53 to 1.44) |  |
| Ⅱ | 49 (24.9) | 19 (18.4) | 1.46 (0.81 to 2.65) |  |
| Ⅲ | 24 (12.2) | 16 (15.5) | 0.75 (0.38 to 1.49) |  |
| Time to successful catheterization (s) (*n*=300) | 141 [115 to 195] | 142 [107 to 202] | 14.2 (-27.8 to 28.0) | 0.943 |
| Total complications (*n*=308) | 33 (16.2) | 3 (2.9) | 6.50 (1.94 to 21.7) | 0.001 |
| Arterial puncture | 1 (0.5) | 2 (1.9) | 0.25 (0.02 to 2.80) | 0.264 |
| Venous hematoma | 33 (16.2) | 1 (1.0) | 19.9 (2.68 to 147) | <0.001 |
| on ultrasound | 31 (15.2) | 1 (1.0) | 18.5 (2.48 to 137) | <0.001 |
| Visible on skin | 17 (8.3) | 0 (0.0) | - | 0.002 |
| Pneumothorax | 0 (0.0) | 0 (0.0) | - | - |
| Hemothorax | 0 (0.0) | 0 (0.0) | - | - |

Values are presented as number (proportion) or median [IQR].

*P* values are the results of Mann-Whitney U test for continuous variable or the chi-square test or Fisher’s exact test according to their expected counts.
